# Analysis of the Efficacy and Safety of Coronary Catheterization through Distal Transradial Access: A Single-Center Data

**DOI:** 10.1155/2023/2560659

**Published:** 2023-05-16

**Authors:** Huanhuan Wang, Dan Liu, Jidong Guo, Nuerbahati Heisha, Lei Wang, Qiang Zhang, Yihui Han, Xiping Wang, Bo Zhang, Jinqing Yuan, Lijian Gao

**Affiliations:** ^1^National Clinical Research Center for Cardiovascular Disease, State Key Laboratory of Cardiovascular Disease, Fuwai Hospital, National Center for Cardiovascular Diseases, Chinese Academy of Medical Sciences and Peking Union Medical College, Beijing, China; ^2^Shihezi People's Hospital, The Third Affiliated Hospital of Shihezi University School of Medicine, Xinjiang, China; ^3^Yunnan Fuwai Cardiovascular Hospital, Kunming, China

## Abstract

**Background and Aims:**

The distal transradial access (dTRA) is a new puncture site for coronary catheterization. We sought to evaluate the feasibility, safety, and complication rates of using the dTRA for cardiac catheterization in Chinese patients.

**Methods:**

A total of 263 consecutive patients who underwent catheterization through the dTRA were enrolled. The primary endpoint of the study was the rate of conversion to another access site due to the impossibility of successful artery puncture or intubation. Secondary safety endpoints were the rates of bleeding-related complications and nerve disorders.

**Results:**

Among 263 patients, the puncture success rate was 96.2% (253/263). Eleven patients were successfully punctured, but the guide wire was difficult to advance. One patient had intubation failure, and the success rate of intubation was 91.6% (241/263). Two hundred thirty-three patients underwent puncture via the right dTRA, 5 patients underwent puncture via the left dTRA, and 3 patients underwent puncture via the bilateral dTRA. A total of 158 (65.6%) patients underwent coronary angiography, and 83 (34.4%) patients underwent percutaneous coronary intervention. After the procedure, only 2 (0.8%) patients had mild bleeding at the puncture site, 2 (0.8%) had a forearm hematoma, and no patient had a nerve disorder.

**Conclusions:**

DTRA has a low incidence of complications, making it a safe and effective technique for cardiac catheterization.

## 1. Introduction

Compared with the femoral artery approach, the transradial artery approach (TRA) for coronary catheterization (CC) is more advantageous [1] in that it reduces the incidence of complications related to the approach site, causes less discomfort in early walking, and reduces mortality in patients with ST-segment elevation myocardial infarction. Therefore, TRA has been recommended as a standard method for CC in the latest European Society of Cardiology (ESC) guidelines [2]. Recent studies have proposed that compared to the classic radial artery access, distal transradial access (dTRA) may be a feasible and safe access site for diagnostic and interventional coronary procedures because of its low incidence of RAO and short time required to achieve hemostasis [3–6]. However, the area of the distal radial artery (RA) is small, and the diameter of the distal RA lumen is small, making it difficult to puncture. Data on the dTRA technique in Chinese populations are limited. Therefore, the aim of this study is to analyze the efficacy and safety of CC through the dTRA in a Chinese population.

## 2. Methods

### 2.1. Study Population

**Figure 1:**
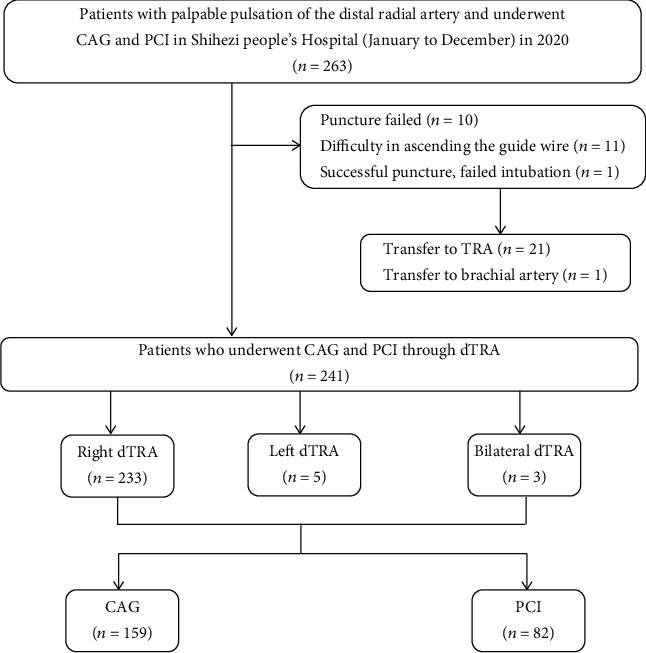
Flowchart of the study.

This was a single-center prospective study of patients who were hospitalized in the Department of Cardiology of Xinjiang Shihezi People's Hospital from January to December 2020. All patients had a palpable pulse at the distal RA in the snuffbox area (Figure 1) and were scheduled to undergo coronary angiography (CAG) and percutaneous coronary intervention (PCI) through the dTRA first. To avoid CC failure due to catheters that were too short, patients who were taller than 180 cm were excluded. Two hundred sixty-three consecutive patients were enrolled (Figure 1). Patients signed informed consent forms, and the study was reviewed by the Ethics Committee of Shihezi People's Hospital (2020LL07-1501, Shihezi, Xinjiang, China).

### 2.2. Procedural Details

**Figure 2:**
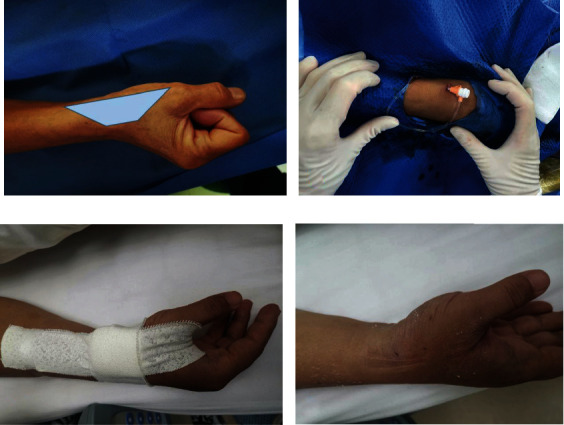
(a) The patient formed a fist to fully expose the anatomical snuffbox (AS) area, which helped us choose the site with the strongest pulse for puncture. (b) Successful cannulation. (c) Bandage compression to stop bleeding. (d) The puncture site after removing the bandage.

The “anatomical snuffbox “ is an area marked by a triangular depression of the abductor pollicis longus tendon, extensor pollicis longus tendon, extensor pollicis brevis tendon, and radial stem [7]. The patient formed a fist to fully expose the anatomical snuffbox (AS) area, which helped us choose the site with the strongest pulse for puncture (Figure 2(a)). After we saw that the blood had returned, the guide wire was inserted smoothly, and then, the sheath was inserted. After CAG and PCI (Figure 2(b)), the sheath was removed, and compression was applied by using gauze pads and elastic bandages (Figure 2(c)). Every 2 hours, the nurse checked whether the compression site had stopped bleeding. If no bleeding or hematoma was found, the elastic band was loosened appropriately. The compression bandage was released after 4 hours (Figure 2(d)). If the puncture site had bleeding or a mild hematoma, the site was pressed for another 30 to 60 minutes.

The operators are familiar with the anatomical structure of the hand arteries, nerves, muscles, and bones and have extensive experience with TRA or dTRA puncture and CC procedures.

### 2.3. Endpoints and Definitions

The primary endpoints of the study were the rates of success puncture and cannulation through the dTRA. The secondary endpoints were the number of puncture attempts, duration of puncture, and time of hemostasis via compression. The safety endpoints were the incidences of bleeding-related complications at the access site and nerve disorders. Minor bleeding and major bleeding were categorized as type 1 or 2 and type 3 or 5, respectively, according to the Bleeding Academic Research Consortium (BARC) [8]. Nerve disorders included finger paresthesia and dysfunction.

### 2.4. Statistical Analysis

Statistical analysis was performed using SPSS 22.0 statistical software. The measurement data are described as the mean ± standard deviation (*x* ± *s*), and the count data are expressed as a percentage (%).

## 3. Results

### 3.1. Baseline Patient Characteristics

Among the 263 patients, 175 (66.5%) were male, with an average age of 64 ± 11 years. Twenty-four (9.1%) patients had previously undergone PCI therapy, and 3 (0.1%) patients had previously undergone coronary artery bypass grafting (CABG) surgery. More than 95% of patients took dual antiplatelet medicines orally (Table 1).

### 3.2. Procedural Characteristics

**Figure 3:**
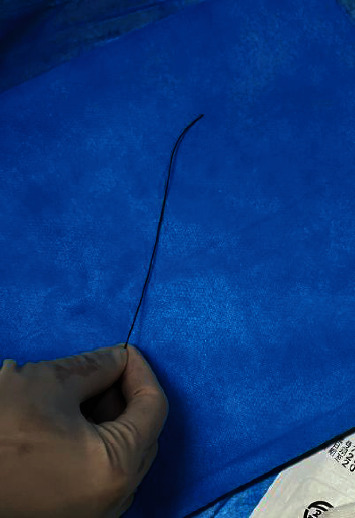
The guide wire was reshaped according to anatomy of the blood vessel.

Among the 263 patients, 10 had failed punctures, leading to a puncture success rate of 96.2% (253/263); 11 patients had successful punctures but had difficulty with guide wire insertion; and 1 patient had failed cannulation. Cannulation was successful in 241 patients, and the rate of successful cannulation through the dTRA was 91.6% (241/263). Among the patients with failed cannulation, 21 patients required conversion to conventional TRA, and 1 patient required conversion to the brachial artery access (Table 2). Difficult guide wire insertion is considered to be related to twisted blood vessels and a lack of clinician experience. The guide wire was reshaped according to the anatomy of the blood vessel (Figure 3), which can increase the rate of successful cannulation.

### 3.3. Procedural and Postprocedural Characteristics of dTRA

Among the 241 patients whose cannulation was successful, 233 patients underwent puncture through the right dTRA, 5 patients through the left dTRA, and 3 patients through the bilateral dTRA; the average number of puncture attempts was 1.2 ± 0.5, and the average puncture duration was 1.7 ± 1.5 minutes. Among the successfully intubated patients, 158 patients underwent CAG, and 83 underwent PCI. The average CAG duration was 12.7 ± 13.5 minutes. In one patient, the occluded RA was accessed and treated through the dTRA. A total of 83 patients (34.4%, 83/241) underwent PCI. A 6F sheath was used to complete all the PCIs. The average compression time was 4.1 ± 1.5 hours. Two (0.4%) patients suffered from mild bleeding after the procedure and 2 (0.4%) had forearm hematomas; there were no cases of severe bleeding or nerve disorders (Table 3).

## 4. Discussion

The aim of this single-center, retrospective study was to observe and analyze the safety and efficacy of CC through the dTRA in a Chinese population. The results show that dTRA has high rates of successful puncture and sheath insertion. CAG and PCI are safe and effective through the dTRA. The compression time of the puncture site was short, and the incidence of bleeding-related complications and nerve disorders was low.

The TRA approach is still the first choice for PCI despite its disadvantages, including radial artery occlusion (RAO), radial nerve injury, radial artery spasm, and puncture complicated by hematoma [9]. In recent years, a new interventional route has been developed; the dTRA is an interventional technique that is used for puncturing the branch of the RA near the root of the thumb, also called snuffbox puncture. This approach allows convenient positioning and efficient hemostasis after the procedure and causes less damage to the proximal RA [10]. In this study, only 2 patients had mild bleeding after dTRA puncture, and 2 had a forearm hematoma. The incidence of bleeding-related complications was extremely low.

RAO is a major problem associated with TRA. Some researchers have shown that the incidence of acute RAO ranges from 1% to 30% [10–12]. In addition, the failure rates of the second puncture and cannulation using the same radial artery were 3.5% (male) and 7.9% (female) [13]. Some single-center research studies and meta-analyses have shown that RAO occurs in only approximately 1.5% of punctures involving the dTRA, which is significantly lower than that of the TRA [14, 15]. Recently, Tsigkas et al. conducted a randomized controlled study [16]. One thousand forty-two consecutive patients were randomized (1 : 1) to the right dTRA or TRA so that the efficacy and safety of the dTRA versus the TRA for CC could be compared. The primary endpoint was the rate of RAO, which was evaluated by Doppler ultrasound 60 days after randomization. The results showed that the rate of RAO was significantly lower in the dTRA group than in the TRA group (3.7% vs. 7.9%, respectively; *P* = 0.014). The lower rate of RAO may have been the result of the anatomical structure of the “snuffbox area.” The snuffbox area is a hollow space bounded by two tendons (extensor longus and extensor brevis). The distal RA is shallow, and the bottom surface is a platform. Bleeding can be stopped by gentle pressure [17]. In this study, 24 patients had previously undergone PCI, and 1 patient had RAO, which was confirmed by distal radial angiography. The incidence of RAO was 4.2%. Retrograde recanalization of the RAO occurred through the dTRA. The dTRA has not only been associated with a reduced incidence of RAO but also a point for recanalization of an RAO, thereby eradicating it.

The diameter of the distal RA lumen is smaller than that of the RA, and whether it is possible to perform complex PCI or use a large-sized sheath remains unclear. However, some studies have shown that the dTRA can not only tolerate large-sized sheaths but can also allow complex PCI procedures. In the Colletti et al. study, 20 patients underwent complex PCI using a 7-French guiding catheter [18]. Multiple bifurcation techniques and calcified plaque-modifying tools were used, the success rate of the procedure was 95%, and no access-site crossover was needed (0%). Only 1 patient experienced spasm. In Asia, Lee et al. reported that 106 patients were treated with bifurcation PCI via the left dTRA [19]. Eleven patients (10.4%) were treated with left main bifurcation, and 39.6% of patients underwent true bifurcations, with the left anterior descending artery/diagonal branch being the most frequent bifurcation site (57.5%, 61/106). PCI was performed using a 6-French guiding catheter in 101 (95.3%) patients and a 7-French guiding catheter in 2 (1.9%) patients. In all 106 patients, PCI via the left dTRA successfully treated bifurcation lesions with no need for access-site crossover. There was no major bleeding, distal and forearm radial artery occlusion, forearm hematoma, or mortality at 30 days. The left DRA is a safe and feasible alternative access site for bifurcation PCI in selected patients. Some studies have shown that vascular ultrasound can significantly increase the success rate of puncture and catheterization, thus preventing multiple puncture attempts and increasing the rate of successful puncture and reducing the risks of hematoma formation and RAO [20–22]. In our study, a 6-French guiding catheter was used in all PCIs, and only a few patients underwent vascular ultrasound examination. Whether the diameter of the distal RA in Chinese patients can tolerate a larger sheath and allow complex PCI still needs the support of more data in the future.

Although the dTRA is advantageous in that it has a lower incidence of RAO, allows faster hemostasis, and is more comfortable for doctors and patients than the TRA, it is also disadvantageous in that it requires more punctures and a longer time for sheath insertion and has a much higher dose area product [16]. These disadvantages may be avoided by using a more advanced device. In any case, the dTRA is an alternative puncture site. It may be the best access point for some selected patients.

## 5. Limitations

This study has many limitations. First, this is a single-center prospective study with a small sample of patients and long follow-up. Second, there was no comparative analysis of the TRA group. Third, a vascular ultrasound examination would have shown that the lumen size could better determine whether the distal RA could bear a larger sheath. Fourth, the time to achieve hemostasis was 4 hours in this study. Due to lack of experience in the early stage, the duration of hemostatic compression was slightly longer. In subsequent studies, we will shorten the duration of hemostatic compression to 1-2 hours and report the occurrence of significant bleeding complications. To date, trials on CAG and PCI via the dTRA in the snuffbox area are underway. Whether dTRA can replace TRA remains to be verified by additional large-scale clinical studies.

## 6. Conclusions

DTRA has been associated with a low incidence of complications and is therefore considered a safe and effective technique for cardiac catheterization.

## Figures and Tables

**Table 1:** Clinical characteristics.

	*N* = 263
*Demographic characteristics*	
Age (years)	64 ± 11
Male sex (%)	175 (66.5)
Mean BMI (kg/m^2^)	25.9 ± 3.4
*Coexisting conditions*	
Hypertension (%)	166 (63.1)
Diabetes mellitus (%)	97 (36.9)
Dyslipidemia (%)	86 (32.7)
Current smoking (%)	60 (22.8)
Family history (%)	6 (2.3)
Previous PCI (%)	24 (9.1)
Previous CABG (%)	3 (0.1)
CVD (%)	89 (33.8)
Ccr < 60 mL/min 1.73 m^2^ (%)	33 (12.5)
*Clinical presentation*	
ACS (%)	164 (62.4)
Stable angina (%)	65 (24.7)
Silent ischemia (%)	22 (8.4)
Non-CAD (%)	12 (4.6)
*Antithrombotic drugs*	
Aspirin (%)	260 (98.9)
Clopidogrel (%)	251 (95.4)
Ticagrelor (%)	1 (0.4)
Tirofiban (%)	2 (0.8)
Bivalirudin	49 (15.6)
Warfarin	1 (0.4)

BMI: body mass index; PCI: percutaneous coronary intervention; CABG: coronary artery bypass grafting; CVD: cerebral vascular disease; Ccr: creatinine clearance; ACS: acute coronary syndrome; CHD: coronary artery disease.

**Table 2:** Procedural characteristics.

	*N* = 263
Change to conventional radial artery access (%)	21 (8.0)
Change to brachial artery (%)	1 (0.4)
Puncture failed (%)	10 (3.8)
Difficulty in ascending the guide wire (%)	11 (4.2)
Successful puncture, failed cannulation (%)	1 (0.4)
Successful dTRA puncture (%)	253 (96.2)
Successful dTRA cannulation (%)	241 (91.6)

dTRA: distal transradial access.

**Table 3:** Procedural and postprocedural characteristics of puncture through the dTRA.

	*N* = 241
Successful dTRA puncture site	
Left (%)	5 (2.1)
Right (%)	233 (96.7)
Bilateral (%)	3 (1.2)
Puncture attempt (times, *x* ± *s*)	1.2 ± 0.5
Puncture durations (min, *x* ± *s*)	1.7 ± 1.5
Coronary angiography time (min, *x* ± *s*)	12.7 ± 13.5
Type of intervention	
CAG (%)	158 (65.6)
PCI (%)	83 (34.4)
Sheath size	
5F (%)	156 (64.7)
6F (%)	85 (35.3)
Coronary angiography time (min, *x* ± *s*)	12.7 ± 13.5
Compression time (h, *x* ± *s*)	4.1 ± 1.5
Open occluded radial artery, *n* (%)	1 (0.4)
Bleeding adverse events, *n* (%)	
Minor bleeding, *n* (%)	4 (1.5)
Mild bleeding, *n* (%)	2 (0.8)
Forearm hematoma, *n* (%)	2 (0.8)
Major bleeding, *n* (%)	0
Nerve disorder, *n* (%)	0

dTRA: distal transradial access; CAG: coronary angiography; PCI: percutaneous coronary intervention.

## Data Availability

The data that support the findings of this study are available on request from the corresponding authors. The data are not publicly available due to privacy or ethical restrictions.

## Abbreviations

TRA: Transradial access
CC: Coronary catheterization
dTRA: Distal transradial access
RA: Radial artery
CAG: Coronary angiography
PCI: Percutaneous coronary intervention
CAD: Coronary artery disease
CABG: Coronary artery bypass grafting
RAO: Radial artery occlusion.
